# Enhanced Intracellular Reactive Oxygen Species by Photodynamic Therapy Effectively Promotes Chemoresistant Cell Death

**DOI:** 10.7150/ijbs.66602

**Published:** 2022-01-01

**Authors:** Xiaolin Xu, Chenglong Wang, Peipei Zhang, Xuzhu Gao, Wencai Guan, Fanchen Wang, Xin Li, Jia Yuan, Hongjing Dou, Guoxiong Xu

**Affiliations:** 1 Research Center for Clinical Medicine, Jinshan Hospital, Fudan University, Shanghai 201508, China.; 2 Department of Oncology, Shanghai Medical College, Fudan University, Shanghai 200032, China.; 3 State Key Laboratory of Metal Matrix Composites, School of Materials Science and Engineering, Shanghai Jiao Tong University, Shanghai 200240, P. R. China.

**Keywords:** Chemoresistance, glycolysis, malignant tumor, oxidative phosphorylation, photodynamic therapy, ROS

## Abstract

Anti-cancer chemo-drugs can cause a rapid elevation of intracellular reactive oxygen species (ROS) levels. An imbalance in ROS production and elimination systems leads to cancer cell resistance to chemotherapy. This study aimed to evaluate the mechanism and effect of ROS on multidrug resistance in various human chemoresistant cancer cells by detecting the changes in the amount of ROS, the expression of ROS-related and glycolysis-related genes, and cell death. We found that ROS was decreased while oxidative phosphorylation was increased in chemoresistant cells. We verified that the chemoresistance of cancer cells was achieved in two ways. First, chemoresistant cells preferred oxidative phosphorylation instead of anaerobic glycolysis for energy generation, which increased ATPase activity and produced much more ATP to provide energy. Second, ROS-scavenging systems were enhanced in chemoresistant cancer cells, which in turn decreased ROS amount and thus inhibited chemo-induced cell death. Our *in vitro* and *in vivo* photodynamic therapy further demonstrated that elevated ROS production efficiently inhibited chemo-drug resistance and promoted chemoresistant cell death. Taken together, targeting ROS systems has a great potential to treat cancer patients with chemoresistance.

## Introduction

Chemotherapy is the main treatment approach for most patients with advanced cancer. However, patients often experience chemoresistance and recurrence 1. The mechanism of the multidrug resistance (MDR) of chemotherapy remains unclear. It has been shown that chemoresistance of cancer is related to many factors, including drug-efflux transport proteins such as P-glycoprotein (P-gp) 2 encoded by ATP-binding cassette subfamily B member 1 (*ABCB1*) gene, and the inhibition of cell apoptosis, etc 3. Chemo-drugs can rapidly elevate intracellular reactive oxygen species (ROS) production (e.g. hydrogen peroxide, superoxide ion, and hydroxyl radicals) 4, which are oxygen derivatives formed in the process of the redox reaction. The elevated ROS can cause cell death, specifically through apoptosis 5, autophagy, ferroptosis, and other death pathways 6. However, to avoid ROS damage, cells eventually initiate ROS-scavenging systems by regulating a series of elimination modulators to remove ROS, such as enhancing the function of the intracellular antioxidant system 7, which leads cancer cells to resist the drug. Some reductive or oxygen-free radical scavenging enzymes such as glucose-6-phosphate dehydrogenase (G6PD) 8, glutamate-cysteine ligase catalytic subunit (GCLC) 9, thioredoxin (TXN) 10, superoxide dismutase (SOD1) 11, NAD(P)H dehydrogenase 1 (NQO1) 12 were involved. Some ROS-induced autophagy and apoptosis molecules such as ferritin light chain (FTL) 13, mucolipin-1 (MCOLN1) 14, hypoxia-inducible factor 1-alpha (HIF1A) 15 may also play a role in chemoresistance.

In general, cancer cells tend to obtain energy through anaerobic glycolysis, which is called the Warburg effect 16 that is thought to be caused by dysfunction of the mitochondria 17, rather than oxidative phosphorylation even in the presence of sufficient oxygen. Some glycolysis-related genes such as glucose transporter-1 (*GLUT1*) 18, hexokinase 2 (*HK2*) 19, pyruvate kinase isozymes M2 (*PKM2*) 20, and glyceraldehyde-3-phosphate dehydrogenase (*GAPDH*) 21, and lactate dehydrogenase A (*LDHA*) 22 may be altered during the chemoresistant processes. Recent studies have also shown that cancer cells have normal mitochondria 23. Cisplatin (DDP)-resistant human ovarian cancer cells tend to mitochondrial oxidative phosphorylation to produce ATP for cell energy supply 24. Therefore, it is essential to understand the mechanisms by which ROS plays a critical role in the regulation of cancer cell survival and the progression of chemoresistance. This study aimed to evaluate the mechanism and the effect of ROS on chemoresistance in cancer cells *in vitro* and *in vivo*.

## Materials and Methods

### Cell lines and cell culture

All cells used in this study were human cancer cells. Ovarian cancer cell line A2780 and its paclitaxel (PTX)-resistant counterpart A2780/PTX (Keygen Biotech, Nanjing, Jiangsu, China), breast cancer cell line MCF-7 (American Type Culture Collection, ATCC, Manassas, VA, USA), its PTX-resistant counterpart MCF-7/PTX and DDP-resistant counterpart MCF-7/DDP (Meixuan Biotech, Shanghai, China) were cultured in DMEM (Gibco, Invitrogen, Carlsbad, CA, USA) supplemented with 10% fetal bovine serum (FBS, Invitrogen). Lung carcinoma cancer cell line A549 (ATCC) and its PTX-resistant counterpart A549/PTX (ATCC), myelogenous leukemia cell line K562 (Keygen Biotech), its doxorubicin (ADR)-resistant counterpart K562/ADR (Keygen Biotech), PTX-resistant counterpart K562/PTX and DDP-resistant counterpart K562/DDP (Meixuan Biotech) were cultured in RPMI-1640 medium (Gibco) supplemented with 10% FBS. The pH value of the culture medium was determined by a pH meter.

### Cell viability and half-maximal inhibitory concentration (IC_50_) detection

Cells were seeded in a 96-well plate at a density of 1×10^4^ cells/well with 100 µL of medium containing various concentrations of chemo-drugs in quadruplicate. The cell viability was detected using the CCK-8 kit (Dojindo, Japan). The optical density (OD) of each well was measured by a microplate analyzer (BioTek Epoch, USA) at 450 nm wavelength. The IC_50_ value was calculated using GraphPad (V5.0).

### ROS detection by flow cytometry

Cells were seeded in a six-well plate at a density of 2×10^5^ cells/well. After incubation for 16 h, cells were treated with or without a chemo-drug at a concentration equal to the IC_50_ for 6 h and conducted for ROS detection. The cells were trypsinized, centrifuged, and stained by using a ROS assay kit (Beyotime Biotechnology, Shanghai, China) according to the product instructions. Subsequently, 500 µL PBS was added to each sample and a ROS level was determined by flow cytometry (Gallios, Beckman Coulter, Inc., Brea, CA, USA). The data were analyzed using FlowJo software (V10, Becton, Dickinson and Company, USA).

### Laser confocal microscopy

Cells were seeded in a 35 diameter mm confocal culture dish with 20 mm of glass-bottom at a volume of 0.5 mL/dish. After confluence reached 60-80%, cells were stained using a ROS assay kit (Beyotime Biotechnology) according to the product instructions, further stained with Hoechst 33342 (Beyotime Biotechnology), washed with PBS for 3 times, and imaged by a laser confocal microscope (SP8 STED 3X, Leica, Germany).

### RNA extraction and quantitative real-time PCR

Total RNA was extracted using an RNA-Quick Purification Kit (ES Science, Shanghai, China) according to the manufacturer's instructions. PCR was applied using a qPCR RT kit (Mei5 Biotechnology, Beijing, China). The PCR primer sequences were shown in Supplementary Table S1. The gene expression levels were normalized to the endogenous control gene 18S and repeatedly to actin to confirm the reliabilities. The threshold cycle (Ct) was determined using the 7300 real-time PCR system (V1.4, Applied Biosystems, USA).

### RNA sequencing analysis

Total RNA was extracted from chemoresistant and chemosensitive cells using an RNA-Quick Purification Kit (ES Science, Shanghai, China) according to the manufacturer's instructions. mRNA sequencing was performed by Gene Denovo Co. Ltd. (Guangzhou, China). In brief, a paired-end library was synthesized using a TruSeq^®^ RNA Sample Preparation Kit (Illumina, USA) according to the preparation guide. After the products were purified, the cDNA libraries were generated by enriching the products with PCR and quantified by the Agilent 2100 bioanalyzer (Agilent Technologies, USA). Molar concentration was determined using a Qubit^®^ 2.0 Fluorometer (Life Technologies, USA). Subsequently, sequencing was performed on an Illumina HiSeq system (Illumina, USA) and the analyses were conducted using HISAT2 (https://daehwankimlab.github.io/hisat2/) and Stringtie (http://ccb.jhu.edu/software/stringtie/) software. Gene Set Enrichment Analysis (GSEA) was performed using the Molecular Signature Database (MSigDB, http://www.gsea-msigdb.org).

### Protein extraction and Western blot analysis

Cells were lysed with sodium dodecyl sulfate (SDS) lysate containing 1% benzyl sulfonyl fluoride and 1% phosphatase inhibitor. After carrying out ultrasonic cracking, the total proteins were run in SDS-polyacrylamide gel electrophoresis. The primary antibodies of the rabbit anti-P-gp (1:50,000 dilution, Cell Signaling Technology, Inc., Boston, MA, USA), pro-apoptotic protein Bax (1:1,000 dilution, Proteintech, Wuhan, China), anti-apoptotic protein Bcl-2 (1:1,000 dilution, Cell Signaling Technology), and mouse anti-β-actin (1:50,000 dilution, Proteintech) and the secondary antibodies of goat anti-rabbit IgG and anti-mouse IgG labeled with horseradish peroxidase (both 1:10,000 dilution, Proteintech) were used. The protein bands were photographed by the chemiluminescence imaging system (Tanon Science & Technology, Shanghai, China).

### Cell death detection

Cells were stained with Annexin-V conjugated with fluorescein (FITC) and propidium iodide (PI) according to the product instructions (BD Pharmingen, BD Biosciences, USA). In brief, cell suspension at a density of 1×10^6^/100 μL was transferred into a 5 mL tube, followed by adding 1 μL of Annexin-V-FITC and/or 3 μL of PI. After incubation with 400 μL of 1× binding buffer in the dark for 15 min, cells were detected by flow cytometry (Gallios, Beckman Coulter, Inc., Brea, CA, USA).

### ATPase activity assay

ATPase activity was determined according to the ATPase kit instructions (Keygen Biotech). Briefly, cells were cultured in a 6-well plate for 24 h. After washing twice with saline, the cells were lysed with a solution containing SDS, followed by ultrasonication. After centrifugation, the suspension was collected. The activity of ATPase was evaluated by measuring the amount of inorganic phosphorus produced by Na^+^K^+^ and Ca^++^Mg^++^ ATPase decomposition. After adding a phosphorus fixing agent for 30 min at 37 ℃, the OD value of each sample was measured by a microplate analyzer at 660 nm wavelength. A 0.5 µmol/ mL of phosphorus storage solution was used for calibration. ATPase activity was calculated according to the following formula: ATPase activity (µ molPi/gHb/hour) = (ODexperment-ODcontrol)/ODcalibrator × Calibrator concentration × Dilution ratio of sample × 6 ÷ Protein concentration.

### *In vitro* photodynamic therapy

Protoporphyrin (Macklin, Shanghai, China) was dissolved in dimethylsulfoxide (DMSO) at a concentration of 4 mg/mL. Cells were seeded in 48-well or 96-well plates (black with clear flat bottom, Corning, USA) and incubated for 24 h. After replacement of fresh medium containing 0~5 mg/mL of protoporphyrin and incubation for 4 h, the cells were irradiated using a laser device (Lei Ze Electronic Technology, Xian, China) with a wavelength at 638 nm, 300 mW/cm^2^ for 0-120 sec. Subsequently, the ROS concentrations were detected using a ROS assay kit, cell viability was determined using a CCK-8 kit, and apoptotic cells were measured using an Annexin-V FITC apoptosis detection kit.

### Establishment of tumor-bearing mice and *in vivo* photodynamic therapy

The ethics of animal experiments was approved by the Ethics Committee of Shanghai Public Health Clinical Center. Four-week-old female BALB/C nude mice were purchased from B&K Laboratory Animals Co., LTD. (Shanghai, China) and raised in a standard feeding condition with adequate food and water. After 7 days, each mouse was injected with approximately 3×10^6^ A549/PTX cells in 100 µL of serum-free RPMI-1640 medium on the right side. After 2 weeks, the tumor-bearing mice were randomly divided into 2 groups (7 per group). Each mouse was injected intratumorally with either 100 µL of normal saline (control) or 100 µL of protoporphyrin (0.3 mg/mL in normal saline) once every 2 days for a total of 5 times. The body weight and tumor volume were monitored during the treatment. The calculation formula of tumor volume is *V = ab^2^/2*, where *V*, *a*, and *b* indicate the volume, tumor length, and tumor width, respectively. At the end of the treatment, the mice were sacrificed and their tumors were excised and photographed. Detection of the production of hydroxyl radicals in tumor tissue lysates was performed according to the hydroxyl radicals production kit instructions (Keygen Biotech). The tumor tissue was fixed with formalin and paraffin-embedded tumor tissue specimens were conducted for hematoxylin-eosin (H&E), proliferating cell nuclear antigen (PCNA), and Tunel assays at Shenghua Biological Technology Co., LTD (Shanghai, China).

### Statistical analyses

The experimental data are shown as mean ± standard deviation (SD) for cell viability, relative fluorescence intensity, and ATPase activity, and as the mean ± standard error (SEM) for IC_50_ values and qRT-PCR. Animal statistics for analyzing the difference between two groups were performed using Student's *t*-test. Statistical results with P < 0.05 were considered statistically significant.

## Results

### ROS level was lower in chemoresistant cells than chemosensitive cells

**Figure 1:**
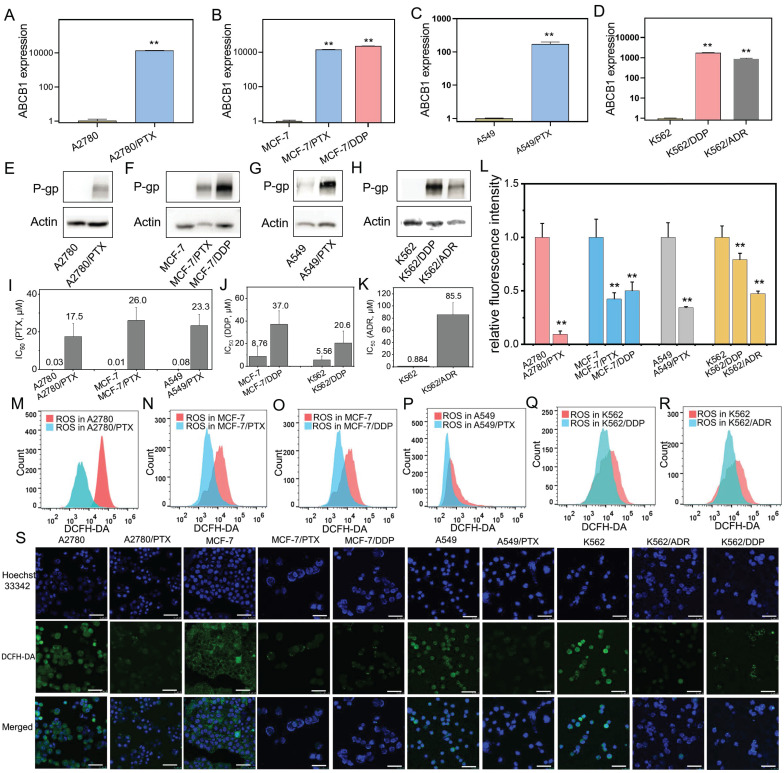
Confirmation of chemoresistant cells and detection of ROS product in different cancer cell lines. (**A-D**) ABCB1 expression in chemoresistant cells (A2780/PTX, MCF-7/PTX, MCF-7/DDP, A549/PTX, K562/DDP, and K562/ADR) and their sensitive counterparts determined by qRT-PCR. The gene expression levels were normalized to the endogenous control gene 18S. (**E-H**) P-gp expression in chemoresistant cells and their sensitive counterparts determined by Western blot. (**I-K**) Measurement of half-maximal inhibitory concentration (IC_50_) in chemoresistant cells and their sensitive counterparts. (**L-R**) Detection of ROS in chemoresistant cells and their sensitive counterparts determined by flow cytometry. Fluorescence intensity represents the level of ROS product. (**S**) Confocal microscopy images of ROS (green) in chemoresistant cells and their sensitive counterparts. The scale bars represent 50 μm. N = 3; **, P < 0,01 (chemoresistant cells *vs*. their chemosensitive cells).

A variety of human chemoresistant cells including ovarian cancer A2780/PTX, breast cancer MCF-7/PTX and MCF-7/DDP, lung cancer A549/PTX, leukemia K562/ADR and K562/DDP cell lines, and their sensitive counterparts A2780, MCF-7, A549, and K562 were used. A chemoresistant marker MDR1 was confirmed to be overexpressed in chemoresistant cells at mRNA (*ABCB1*) (Figure 1A-D) and protein (P-gp) levels (Figure 1E-H) compared with their sensitive counterpart cells. The chemoresistant cells were further confirmed by IC_50_ detection. The IC_50_ value was increased in these chemoresistant cells compared with their sensitive counterpart cells (Figure 1I-K).

Next, we found that the ROS levels were lower in chemoresistant cells than chemosensitive cells determined by flow cytometry (Figure 1L-R) and laser confocal microscopy (Figure 1S).

### Chemotherapeutic drugs elevated intracellular ROS levels in both chemoresistant and chemosensitive cells

**Figure 2:**
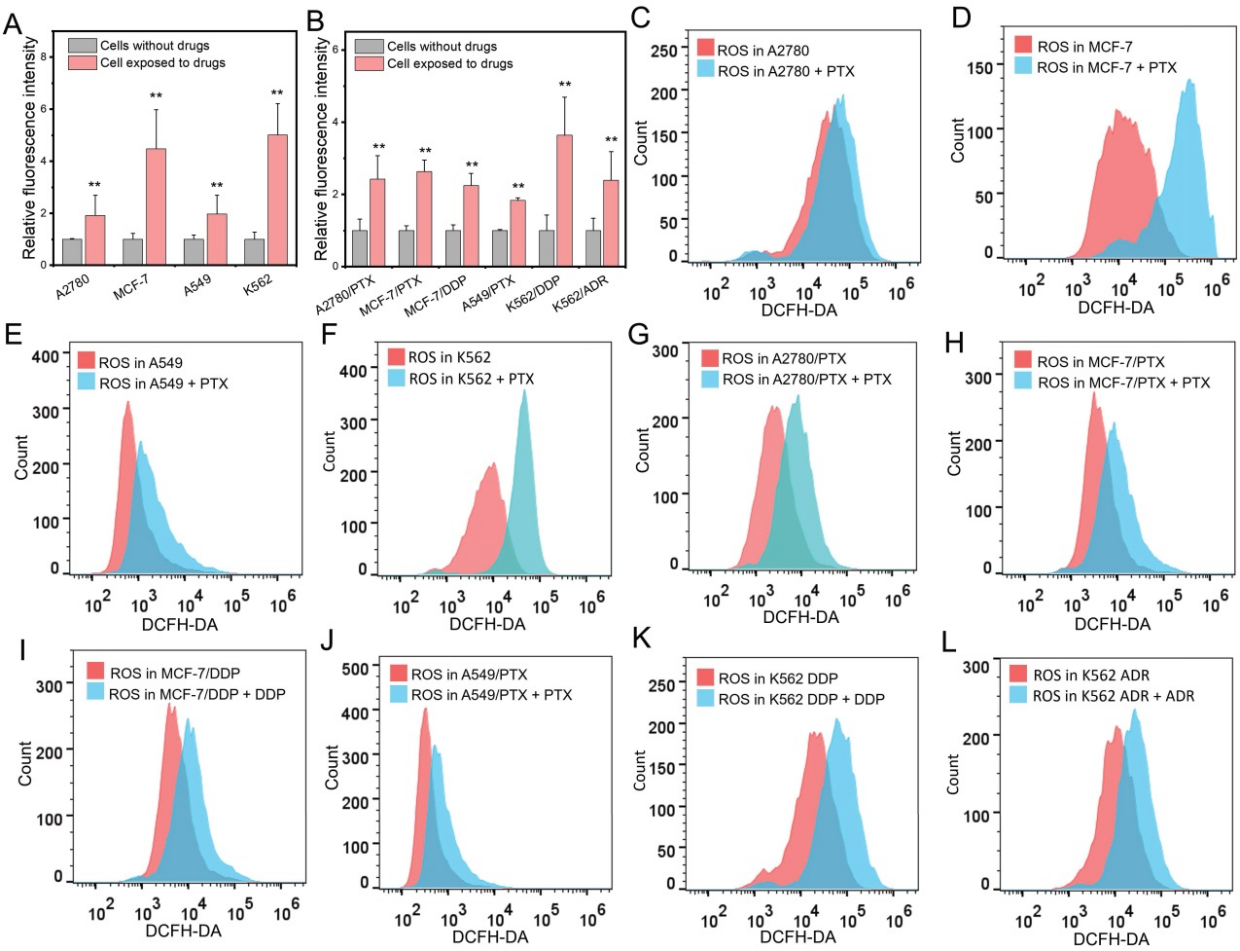
Changes of intracellular ROS after chemotherapeutic drug treatment. (**A, B**) Measurement of ROS amount in chemosensitive and chemoresistant cells exposed to chemotherapeutic drugs. (**C-F**) Detection of ROS level in chemosensitive cells exposed to PTX after 24 h determined by flow cytometry. (**G-L**) Detection of ROS level in chemoresistant cells exposed to PTX (G, H, J), DDP (I, K), ADR (L) for 24 h determined by flow cytometry. The drug concentration was consistent with the corresponding IC_50_. N = 3; ∗∗, P < 0.01 (chemoresistant cells *vs*. their chemosensitive cells).

In chemosensitive cells (SK-OV-3), the levels of ROS were elevated after exposure to a chemotherapeutic drug PTX (Figure S1). Next, we extensively treated chemosensitive and chemoresistant cells with different chemotherapeutic drugs and found that the levels of ROS were elevated in both chemosensitive and chemoresistant cells after drug administration (Figure 2A and B). Further experiments showed that PTX increased ROS concentrations in A2780, MCF-7, A549, and A562 chemosensitive cells (Figure 2C-F). The drug concentration was consistent with the corresponding IC_50_. Interestingly, chemotherapeutic drugs also increased the ROS concentration in chemoresistant cells (Figure 2G-L).

### ROS-scavenging system was enhanced in chemoresistant cancer cells

**Figure 3:**
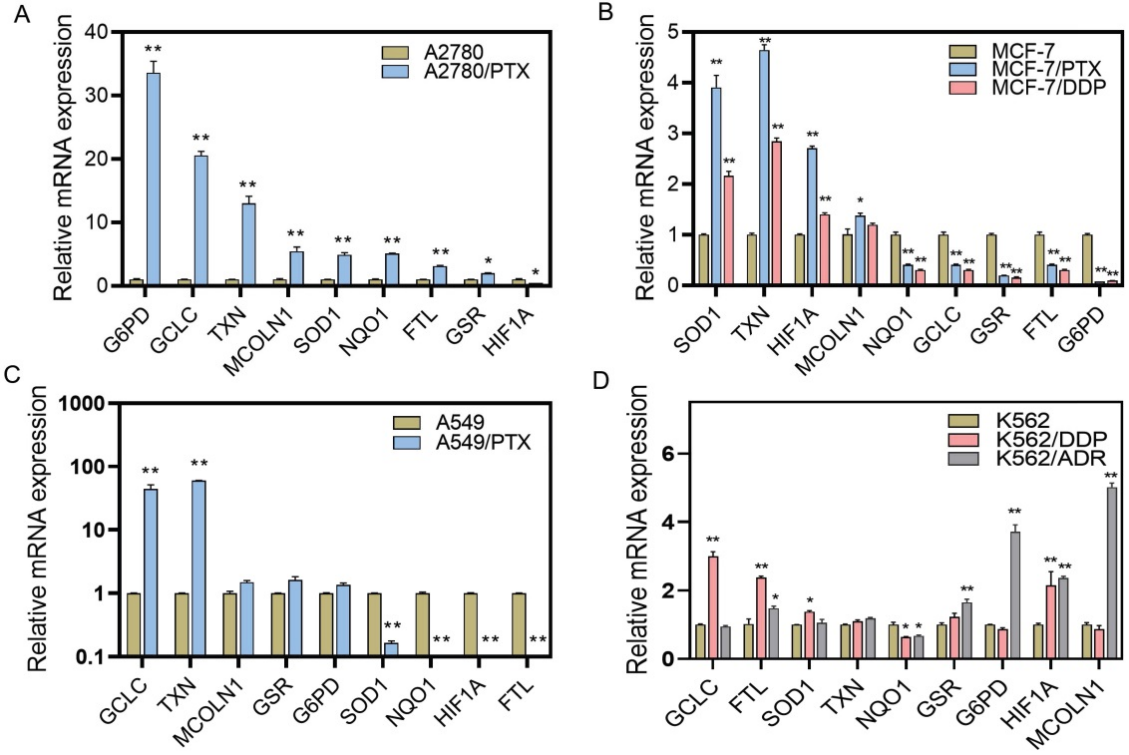
Expression of ROS-related genes in chemoresistant and chemosensitive cancer cells. (**A**) Expression of ROS-related genes in A2780/PTX cells and its sensitive counterpart A2780 cells determined by qRT-PCR. (**B**) Expression of ROS-related genes in MCF-7/PTX, MCF-7/DDP cells, and their sensitive counterpart MCF-7 cells determined by qRT-PCR. (**C**) Expression of ROS-related genes in A549/PTX and its sensitive counterpart A549 cells determined by qRT-PCR. (**D**) Expression of ROS-related genes in K562/DDP, K562/ADR, and their sensitive counterpart K562 cells determined by qRT-PCR. The gene expression levels were normalized to the endogenous control gene 18S. N = 3; ∗, P < 0.05; ∗∗, P < 0.01 (chemoresistant cells *vs*. their chemosensitive cells). FTL, ferritin light chain; G6PD, glucose-6-phosphate dehydrogenase; GCLC, glutamate-cysteine ligase catalytic subunit; GSR, glutathione-disulfide reductase; HIF1A, hypoxia-inducible factor 1-alpha; MCOLN1, mucolipin-1; NQO1, NAD(P)H dehydrogenase 1; SOD1, superoxide dismutase; TXN, thioredoxin.

Because the ROS levels were elevated after chemotherapeutic drug treatment and were lower in chemoresistant cells than chemosensitive cells, we examined the ROS-scavenging system by detecting ROS elimination-related mRNAs using qRT-PCR. We found that the expression of some reductive or oxygen-free radical scavenging enzymes was increased in chemoresistant cells, including *G6PD* in A2780/PTX and K562/ADR cells, *GCLC* in A2780/PTX, A549/PTX, K562/DDP, and K562/PTX cells, *TXN* in A2780/PTX, MCF-7/PTX and MCF-7/DDP, A549/PTX and K562/PTX cells, *SOD1* in A2780/PTX, MCF-7/PTX and MCF-7/DDP, and K562/PTX cells, *NQO1* in A2780/PTX cells (Figure 3A-D, Figure S2). Accordingly, some genes of ROS production, ROS-induced autophagy, and apoptosis were also changed in chemoresistant cells, including *FTL* in A2780/PTX, K562/DDP, K562/ADR and K562/PTX cells, *MCOLN1* in A2789/PTX and K562/ADR cells, *HIF1A* in MCF-7/PTX and MCF-7/DDP, K562/DDP and K562/ADR cells (Figure 3). The expression of *ABCB1* mRNA was confirmed to be increased in K562/PTX resistant cells (Figure S3).

### Glycolysis was inhibited and oxidative phosphorylation was increased in chemoresistant cancer cells

**Figure 4:**
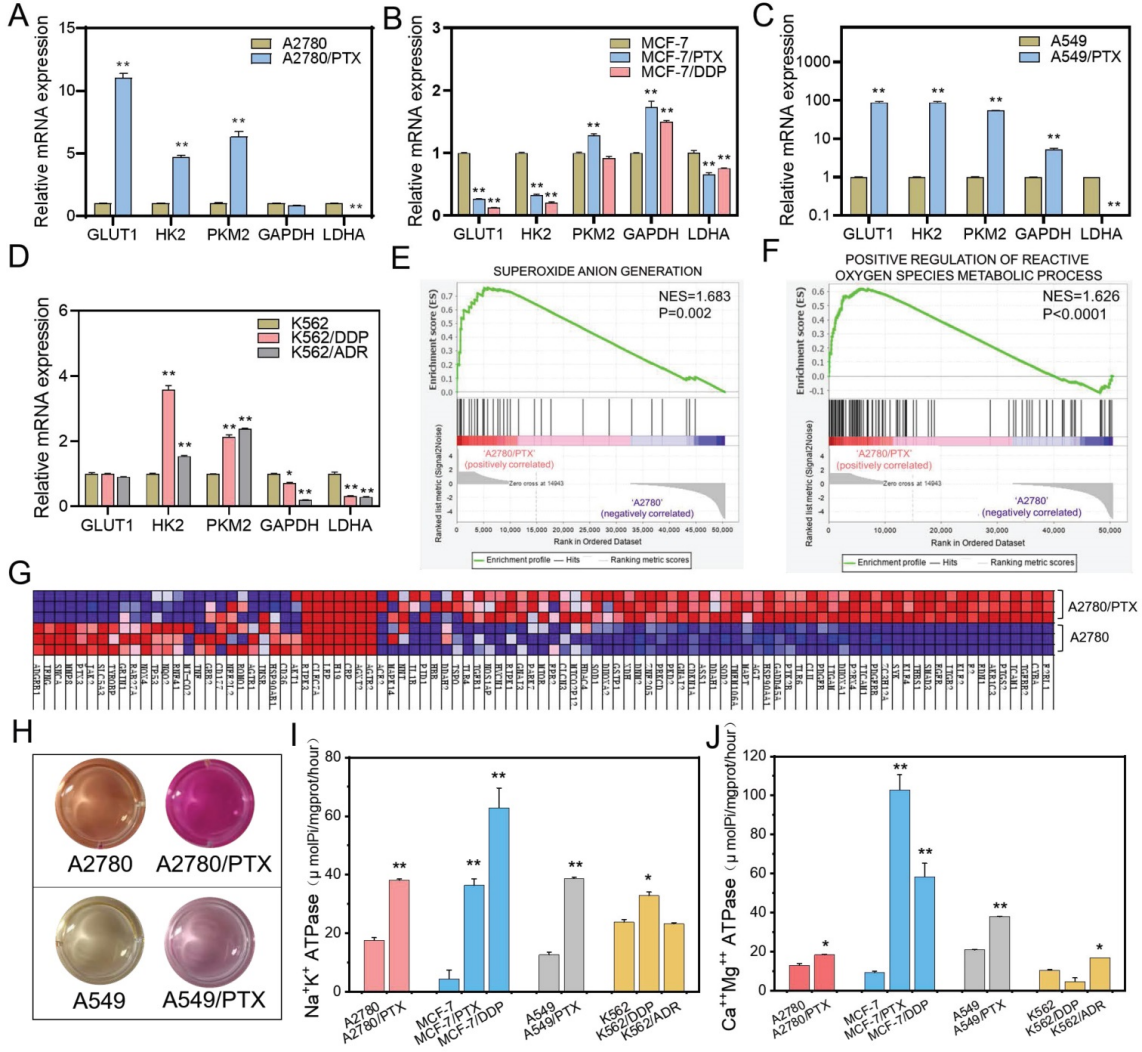
Differences in glucose metabolism between chemoresistant and sensitive cancer cells. (**A-D**) Expression of glycolysis-related genes in chemoresistant cells and their sensitive counterparts as determined by qRT-PCR. The gene expression levels were normalized to the endogenous control gene 18S. (**E**) GSEA revealing enrichment of oxidative phosphorylation gene signature in A2780/PTX cells compared to A2780 cells. (**F**) GSEA revealing enrichment of ATP synthesis-coupled electron transport gene signature in A2780/PTX cells compared to A2780 cells. (**G**) Heatmap of ATP-synthesis coupled electron transport gene signature in A2780/PTX cells compared to A2780 cells. (**H**) Photos of the color changing of the medium after A2780, A2780/PTX, A549, and A549/PTX cells were cultured for 24 h. (**I**) Detection of Na^+^K^+^ ATPase activities in chemoresistant cells and their sensitive counterparts. (**J**) Detection of Ca^++^Mg^++^ ATPase activities in chemoresistant cells and their sensitive counterparts. N = 3; ∗, P < 0.05; ∗∗, P < 0.01 (chemoresistant cells *vs*. their chemosensitive cells).

Generally, cancer cells tend to obtain energy through anaerobic glycolysis rather than oxidative phosphorylation. However, in chemoresistant cells, glycolysis-related mRNAs including *GLUT1*, *HK2*, *PKM2*, and *GAPDH* were slightly upregulated, and *LDHA* were downregulated (Figure 4A-D, Figure S4), which proved that aerobic glycolysis was inhibited in chemoresistant cells. Moreover, the genes related to ROS generation were increased, while the ROS amount was decreased, in chemoresistant cells compared with chemosensitive cells, (Figure 4E-G). In addition, the change of the color of the culture medium indicated weaker acidic in chemoresistant cells (Figure 4H). The pH value of the culture media of A2780, A2780/PTX, A549, and A549/PTX cells were 6.95±0.15, 7.73±0.11, 6.94±0.08, and 7.54±0.28, respectively, after cultivation for 24 h, which demonstrated the restraint of the Warburg effect in chemoresistant cells. Further study also showed that ATPase activity was enhanced in chemoresistant cells compared with chemosensitive cells (Figure 4I-J). These results suggest that chemoresistant cells achieved drug resistance by inhibiting glycolysis and increasing the energy generated by oxidative phosphorylation.

### Photodynamic therapy promoted chemoresistant cell death *in vitro*

**Figure 5:**
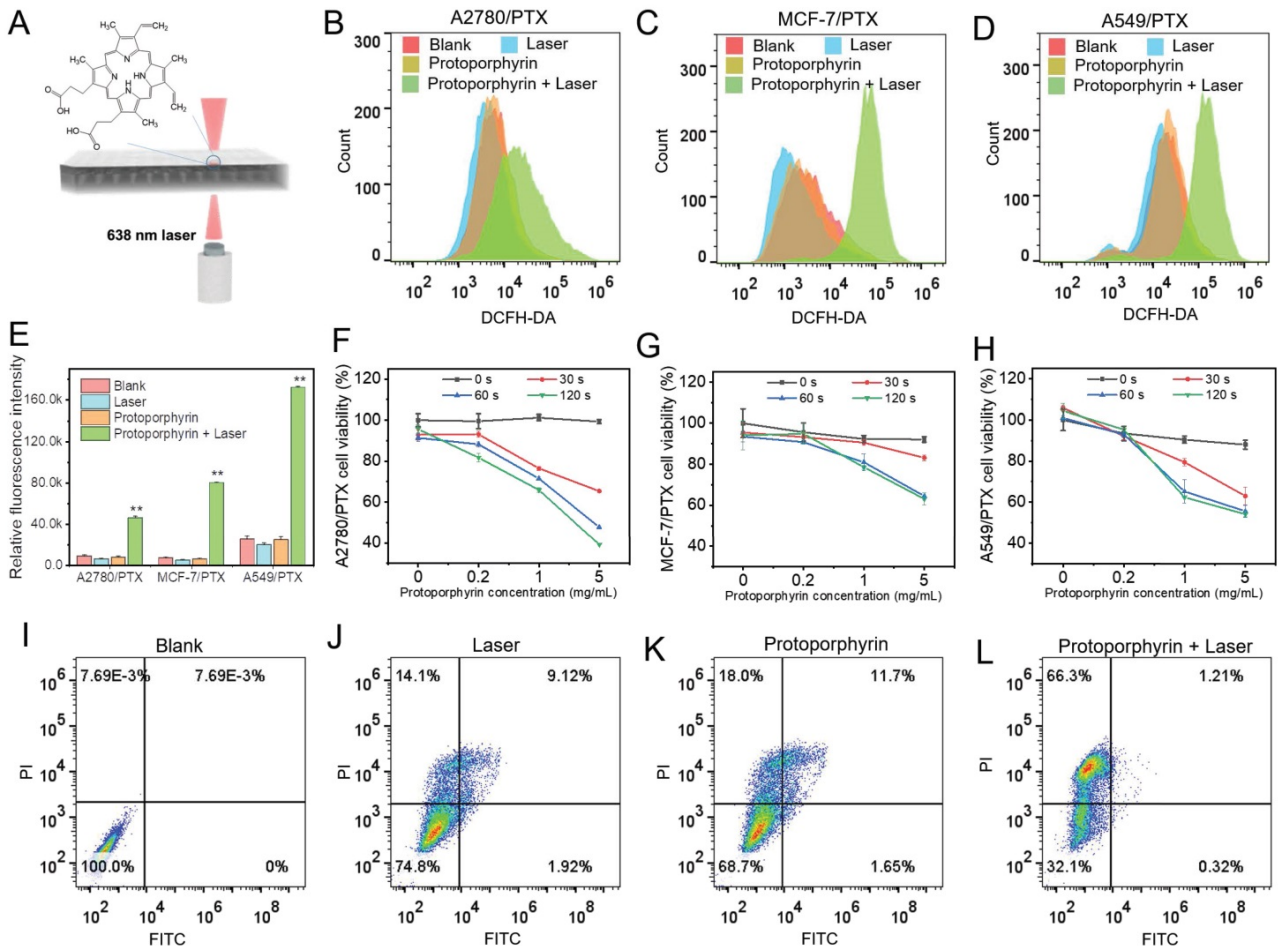
The effect of photodynamic therapy in reversing drug resistance. (**A**) Schematic illustration depicting the *in vitro* photodynamic therapy. (**B-D**) Detection of ROS in A2780/PTX, MCF-7/PTX, and A549/PTX cells measured by flow cytometry after photodynamic therapy. (**E**) Quantitative analysis of ROS amount in A2780/PTX, MCF-7/PTX, and A549/PTX cells. N = 3; ∗∗, P < 0.01 (*vs*. controls). (**F-H**) Cell viability of A2780/PTX, MCF-7/PTX, and A549/PTX cells treated with various concentrations of protoporphyrin for 0, 30, 60, and 120 sec. (**I-L**) Measurement of MCF-7/PTX cells death by flow cytometry after photodynamic therapy.

Next, we used protoporphyrin as a photosensitizer for photodynamic therapy (Figure 5A). As protoporphyrin may be a substrate for ABC transporter such as ABCG2/BCRP 25, next we examined the expression of *ABCG2* in chemoresistant cells. We found that the *ABCG2* level was not increased in most chemoresistant cells compared with their sensitive cells, except for A2780/PTX (Figure S5), indicating that protoporphyrin would be retained in the cytosol and the efficacy of treatment would not be affected by the efflux pump ABCG2 in most chemoresistant cells. Furthermore, the ROS levels in chemoresistant cells were significantly elevated after laser irradiation (Figure 5B-E), while the cell viability of chemoresistant cells was significantly reduced (Figure 5F-H), along with the promotion of cancer cell death (Figure 5I-5L). Flow cytometry data showed that there was a significant difference in the number of dead cells between the Laser (25.14%), Protophyrin (31.35%), and Laser+Protophyrin (67.83%) groups (Figure 5I-5L). However, the apoptotic cell population was lower in the Laser+Protophyrin (1.21+0.32%) group compared to Laser (9.12+1.92%) and Protophyrin (11.7+1.65%) groups. In addition, we observed that the ratio of Bax/Bcl-2 between chemoresistant and sensitive cells was similar when cells were cultured in a chemo-free medium (Figure S6). These data indicate that photodynamic therapy can elevate the level of ROS which may effectively promote chemoresistant cell death but not through apoptosis.

Because *GAPDH* is a glycolysis-related gene and was differentially expressed between chemoresistant and sensitive cells, 18S was applied as an endogenous control gene to normalize the interested genes. To confirm the reliabilities of qRT-PCT, another endogenous control gene *Actin* was applied. Indeed, the results of relative mRNA expression of targeted genes were reproducible after normalizing to Actin. (Figure S7).

### Photodynamic therapy inhibited tumor progression *in vivo*

**Figure 6:**
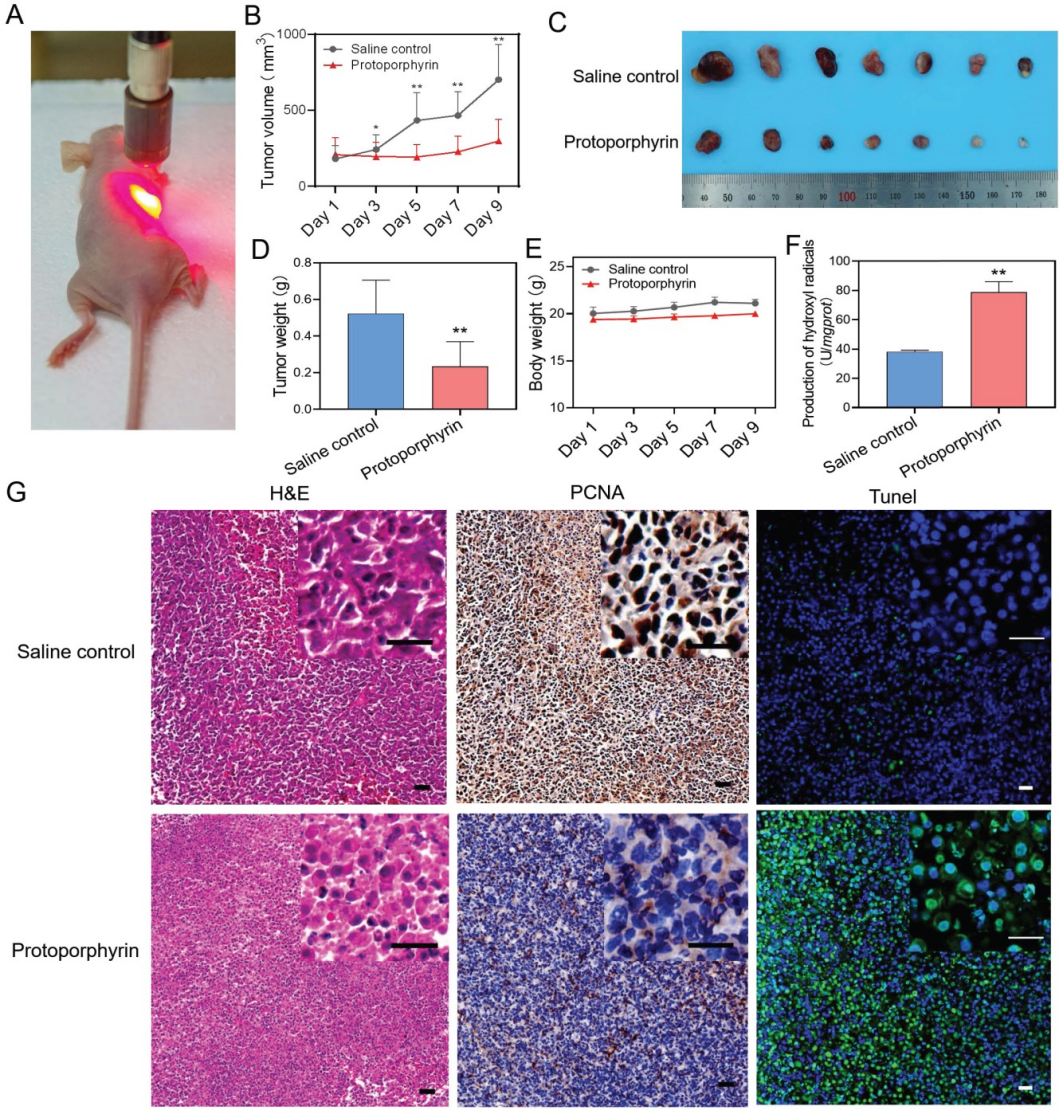
*In vivo* photodynamic therapy in chemoresistant cell bearing mice. (**A**) Photo of *in vivo* photodynamic therapy. (**B**) Tumor volume of the saline control and protoporphyrin groups during treatment. (**C**) Picture of tumors harvested from the saline control and protoporphyrin groups after 9 days of treatment (N = 7). (**D**) Tumor weight of the saline control and protoporphyrin groups after 9 days of treatment. (**E**) Body weight of the saline control and protoporphyrin groups during treatment. (**F**) Detection of the production of hydroxyl radicals in tumor tissue lysates after treatment. (**G**) H&E, PCNA, and Tunel stained tumor slices from the saline control and protoporphyrin groups, respectively. Original magnification ×100, inset magnification ×400. The scale bars represent 20 μm. ∗∗, P < 0.01 (*vs*. controls).

Animal experiments were conducted to evaluate the *in vivo* effect of photodynamic therapy on the inhibition of chemoresistant cancer cells. A549/PTX bearing mice were injected intratumorally with normal saline or protoporphyrin and irradiated with a 638 nm laser (Figure 6A). We found that the group injected with protoporphyrin displayed inhibitory growth of chemoresistant tumors (Figure 6B). The protoporphyrin injection group had a significant therapeutic effect compared with the normal saline injection group (Figure 6C-D) after photodynamic therapy. The body weight of mice was unchanged, indicating the safety of photodynamic therapy (Figure 6E). The ability to produce hydroxyl anions in tumor tissue of the protoporphyrin group was significantly higher than that in the normal saline group (Figure 6F), proving that ROS produced by protoporphyrin plays a long-term effect on tumor progression. H&E, PCNA, and Tunel stained tumor slices showed that tumor necrosis was increased, proliferation was decreased, and cell death was induced in the protoporphyrin group compared with the normal saline group (Figure 6G). All these results indicate that photodynamic therapy can successfully achieve effective treatment for chemoresistant tumors.

## Discussion

Patients with advanced cancer often face a big challenge of chemoresistance during chemo-drug treatment. An imbalance of ROS level may play a critical role in cells resistant to a chemo-drug. The present study examined the value of ROS, the expression of ROS-related genes, and glycolysis-related genes in various chemoresistant cancer cells. Here, we observed interesting results that the intracellular level of ROS was significantly lower in chemoresistant cancer cells than chemosensitive cancer cells, while a chemo-drug elevated the intracellular ROS level in both chemoresistant and chemosensitive cancer cells. It leads to a hypothesis that the decrease of ROS concentration in chemoresistant cells is due to the enhancement of the ROS-scavenging system during chemoresistant processes. Indeed, we confirmed that an increase in the ROS elimination together with the restraint of aerobic glycolysis conducts cancer cell resistance to a chemo-drug.

The current study demonstrated that ATPase activity was enhanced in chemoresistant cells. Unlike chemosensitive tumor cells that mainly rely on glycolysis 26, chemoresistant cells obtain energy mainly through oxidative phosphorylation. Aerobic oxidative phosphorylation can produce a large amount of ATP 27, while anaerobic glycolysis produces a relatively small amount of ATP 28. P-gp is a drug efflux transporter and since P-gp is an ATP-dependent protein 29, 30, more production of ATP by oxidative phosphorylation is conducive to the enhancement of the activity of P-gp and the development of chemoresistance in cancer cells. Overexpression of P-gp leads to chemoresistant cells more capable of invasion and migration 31. It is well known that ROS is mainly produced by oxidative phosphorylation as well 32, 33. Although oxidative phosphorylation produces a large amount of reactive oxygen free radicals, the ROS-scavenging system is also enhanced in chemoresistant cells and thus leads to the reduction of the amount of ROS, which further results in drug resistance of cancer cells.

We did observe an increase of ROS in chemoresistant cells after a chemo-drug treatment, which is consistent with the previous report that ROS was elevated in cisplatin-resistant ovarian cancer cells 34. Given the direct relationship between ROS and drug resistance, increasing intracellular ROS may be an important factor to reverse anti-cancer drug resistance 35. Therefore, targeting ROS systems has a great potential to treat cancer patients with chemoresistance. Thus, reversal of multidrug resistance of cancers can be achieved by the elevation of intracellular ROS levels. It has been shown that photodynamic therapy is an efficient method to produce ROS 36. Our *in vitro* and *in vivo* experiments using photodynamic therapy showed that an increase in ROS can promote chemoresistant cell death. Because protoporphyrins are widely used to produce ROS efficiently 37, we used photodynamic therapy as an effective method to generate ROS and suppress tumor growth.

It has been known that chemoresistant cells can inhibit apoptosis 3. In the current study, the ratio of Bax/Bcl-2 between chemoresistant and sensitive cells was similar when cells were cultured in a chemo-free medium, suggesting that the inhibition of apoptosis in chemoresistant cells was achieved by reducing the intracellular level of ROS rather than by upregulating anti-apoptotic gene expression. In addition, a ROS level was elevated after exposure to chemotherapy drugs in chemosensitive cells, indicating that chemotherapeutic drugs can produce enough ROS to induce apoptosis in chemoresistant cells. Our data further confirmed the previous study that chemoresistant cells may activate the ROS-scavenging system to inhibit apoptosis 35.

Our results showed that chemoresistant cells indeed activated the ROS-scavenging system as the expression of *G6PD*, *TXN*, and *GCLC* was upregulated. This series of genes that constitute the ROS scavenging system indeed lead to the decrease of ROS levels in chemoresistant cells. TXN as a key protein of the thioredoxin system was also significantly increased in the chemoresistant cells, indicating that the thioredoxin system may play an important role in the reduction of ROS in chemoresistant cells. Therefore, increasing intracellular ROS concentration may be a therapeutic strategy to reverse chemoresistance. Thus, photodynamic therapy has a great value to treat cancer patients with chemoresistance.

**Figure 7:**
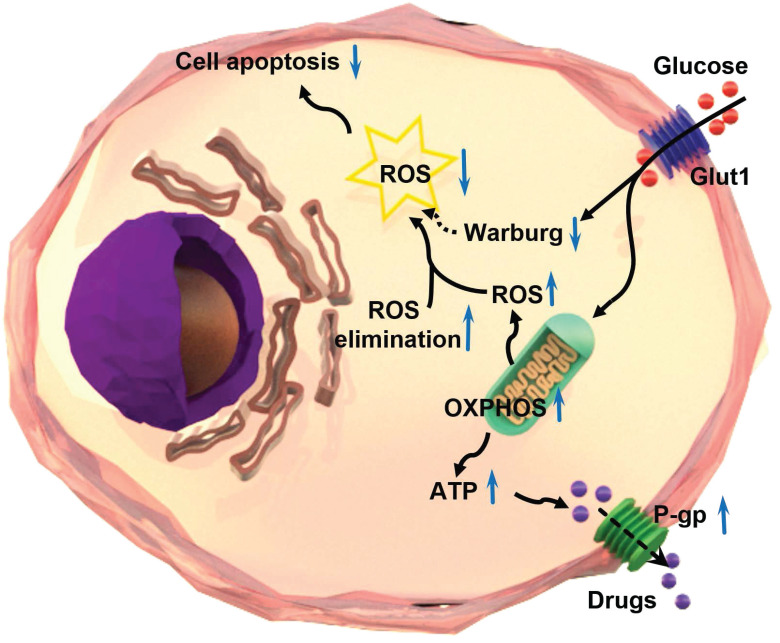
Schematic illustration of the mechanism of multidrug resistance in a chemoresistant cancer cell by increasing oxidative phosphorylation (OXPHOS) and reducing reactive oxygen species (ROS).

In summary, the intracellular level of ROS is significantly lower in chemoresistant cancer cells than chemosensitive cells. Chemoresistant cancer cells prefer oxidative phosphorylation instead of anaerobic glycolysis for energy generation. The ROS-scavenging system is enhanced and aerobic glycolysis is restrained in chemoresistant cells. The reduction of ROS by oxidative phosphorylation results in the inhibition of chemotherapeutic drug-induced apoptosis, and in turn, leads to the progression of chemoresistance in cancer cells (Figure 7). The significance of the findings extends our knowledge of the relationship between ROS production/clearance and chemo-drug resistance and provides a novel mechanistic and technical meaning of reversing chemoresistance by increasing ROS production. Photodynamic therapy is highly effective to produce ROS that has proved to be important in inhibiting the chemoresistance of cancer cells. Thus, targeting ROS systems has clinical potential.

## Supplementary Material

Supplementary figures and table.
