# Supplementary material for: Enhanced Intracellular Reactive Oxygen Species by Photodynamic Therapy Effectively Promotes Chemoresistant Cell Death
Source: Int J Biol Sci. 2022 Jan 1;18(1):374–85. doi: 10.7150/ijbs.66602 (PMC8692137; doi:10.7150/ijbs.66602)
Supplement: Supplementary file 1 — Supplementary figures and table. [file ijbsv18p0374s1.pdf]

Supplementary Materials

Enhanced Intracellular Reactive Oxygen Species by Photodynamic Therapy Effectively Promotes Chemoresistant Cells Death

Xiaolin Xu [1,2,†], Chenglong Wang [1,†], Peipei Zhang [3], Xuzhu Gao [1], Wencai Guan [1], Fanchen Wang [1,2], Xin Li [1,2], Jia Yuan [1,2], Hongjing Dou [3,*], Guoxiong Xu [1,2,*]

[1] Research Center for Clinical Medicine, Jinshan Hospital, Fudan University, Shanghai 201508, China; sdzzxxl@163.com (X.X); wangchenglong1106@126.com (C.W.); alexgwan@163.com (X.G.); napols8@163.com (W.G.); wangfanchen@live.com (F.W.); Micki0228@163.com (X.L.); yj18779116489@163.com (J. Y.); guoxiong.xu@fudan.edu.cn (G.X.)

[2] Department of Oncology, Shanghai Medical College, Fudan University, Shanghai 200032, China; sdzzxxl@163.com (X.X); wangfanchen@live.com (F.W.); Micki0228@163.com (X.L.); yj18779116489@163.com (J. Y.); guoxiong.xu@fudan.edu.cn (G.X.)

[3] State Key Laboratory of Metal Matrix Composites, School of Materials Science and Engineering, Shanghai Jiao Tong University, Shanghai 200240, P. R. China; zhangpeipei@sjtu.edu.cn (P.Z); hjdou@sjtu.edu.cn (H.D.)

* Correspondence: guoxiong.xu@fudan.edu.cn (G.X.); hjdou@sjtu.edu.cn (H.D.); Tel.: +86-21-34189990 (G.X.); +86-21-34202956 (H.D.)

† These authors contributed equally to this work.

Table S1 PCR Primer sequences.

Name		Sequence (5’ → 3’)
ABCB1	Forward	CACATTTGGCAAAGCTGGAGA
	Reverse	CATCATTGGCGAGCCTGGTA
ABCG2	Forward	CAGGTGGAGGCAAATCTTCGT
	Reverse	ACCCTGTTAATCCGTTCGTTTT
ACTIN	Forward	TCATCACCATTGGCAATGAG
	Reverse	CACTGTGTTGGCGTACAGGT
FTL	Forward	CAGCCTGGTCAATTTGTACCT
	Reverse	GCCAATTCGCGGAAGAAGTG
G6PD	Forward	CGAGGCCGTCACCAAGAAC
	Reverse	GTAGTGGTCGATGCGGTAGA
GAPDH	Forward	GCACCGTCAAGGCTGAGAAC
	Reverse	TGGTGAAGACGCCAGTGGA
GCLC	Forward	GGAGGAAACCAAGCGCCAT
	Reverse	CTTGACGGCGTGGTAGATGT
GLUT1	Forward	CATCCCATGGTTCATCGTGGCTGAACT
	Reverse	GAAGTAGGTGAAGATGAAGAACAGAAC
GSR	Forward	CACTTGCGTGAATGTTGGATG
	Reverse	TGGGATCACTCGTGAAGGCT
HIF1A	Forward	GAACGTCGAAAAGAAAAGTCTCG
	Reverse	CCTTATCAAGATGCGAACTCACA
HK2	Forward	GCCATCCTGCAACACTTAGGGCTTGAG
	Reverse	GTGAGGATGTAGCTTGTAGAGGGTCCC
LDHA	Forward	ATGGCAACTCTAAAGGATCA
	Reverse	GCAACTTGCAGTTCGGGC
MCOLN1	Forward	TTCGCCGTCGTCTCAAATACT
	Reverse	CTCTTCCCGGAATGTCACAGC
NQO1	Forward	GAAGAGCACTGATCGTACTGGC
	Reverse	GGATACTGAAAGTTCGCAGGG
PKM2	Forward	GCCCGTGAGGCAGAGGCTGC
	Reverse	TGGTGAGGACGATTATGGCCC
SOD1	Forward	GGTGGGCCAAAGGATGAAGAG
	Reverse	CCACAAGCCAAACGACTTCC
TXN	Forward	GTGAAGCAGATCGAGAGCAAG
	Reverse	CGTGGCTGAGAAGTCAACTACTA

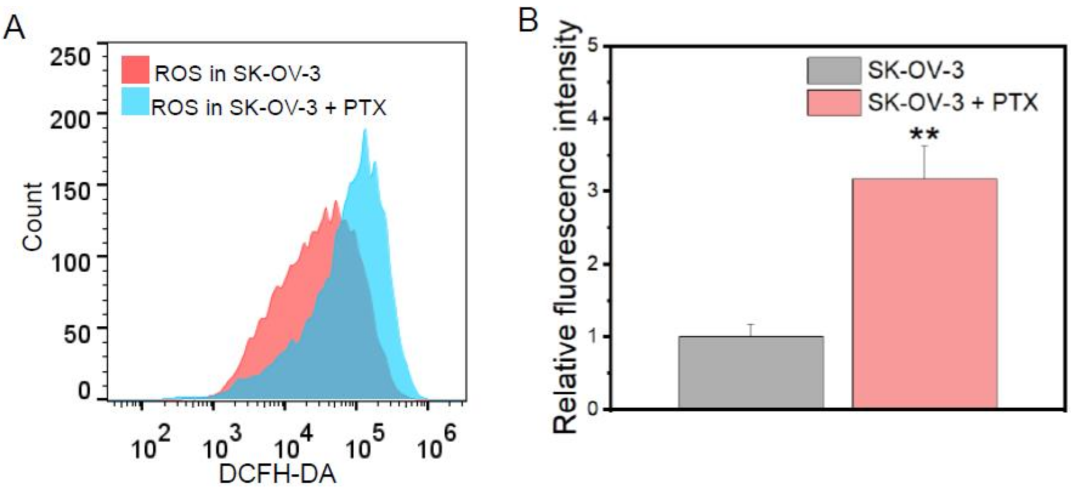

Figure S1. Level of ROS in SK-OV-3 cells before and after exposure to chemotherapeutic drug paclitaxel (PTX) determined by flow cytometry. **(A)** Detection of ROS level in SK-OV-3 cells exposed to PTX determined by flow cytometry. **(B)** Quantitative analysis of ROS amount in SK-OV-3 cells. N= 3; **, P < 0.01 (chemoresistant *vs*. chemosensitive cells).

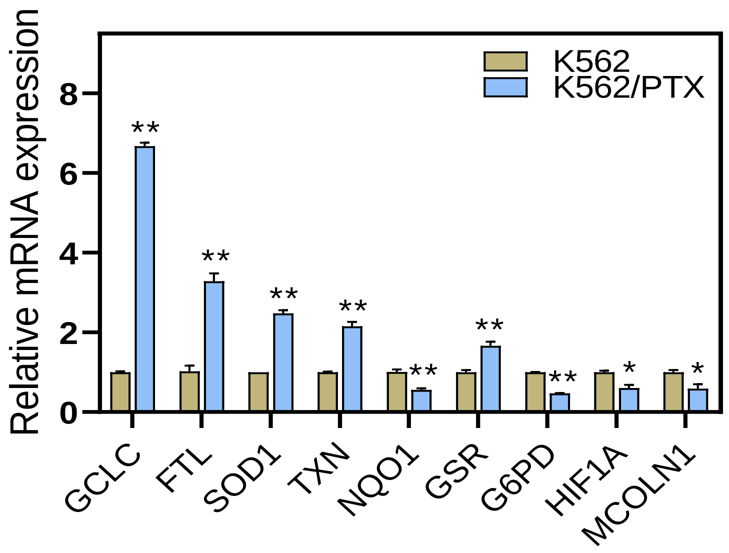

Figure S2. Expression of ROS-related mRNAs in K562 and PTX-resistant K562 cells (K562/PTX) determined by qRT-PCR. The gene expression levels were normalized to the endogenous control gene 18S. N = 3; ∗, P < 0.05; ∗∗, P < 0.01 (chemoresistant *vs*. chemosensitive cells).

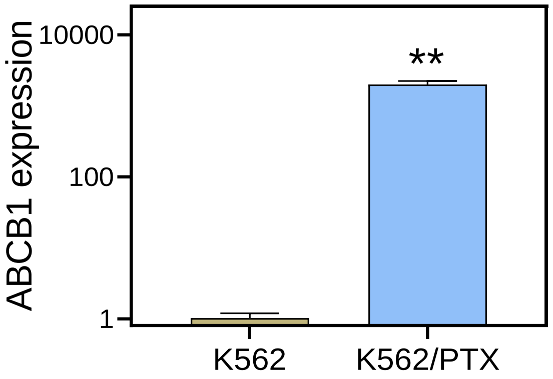

Figure S3. Expression of ABCB1 mRNA in K562 cells and PTX-resistant K562 cells (K562/PTX) determined by qRT-PCR. The gene expression levels were normalized to the endogenous control gene 18S. N = 3; **, P < 0.01 (chemoresistant *vs*. chemosensitive cells).

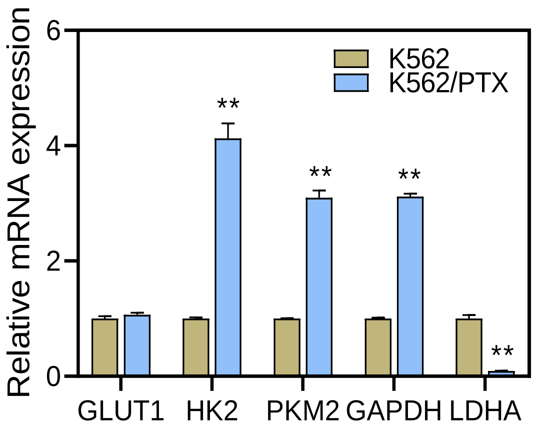

Figure S4. Expression of glycolysis-related mRNAs in K562 and K562/PTX cells determined by qRT-PCR. The gene expression levels were normalized to the endogenous control gene 18S. N = 3; **, P < 0.01 (chemoresistant *vs*. chemosensitive cells).

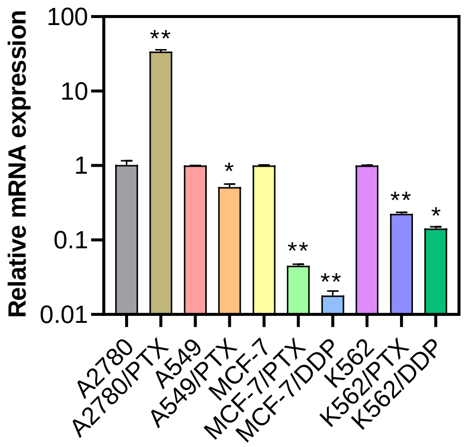

Figure S5. Expression of ABCG2 in chemoresistant cells determined by qRT-PCR. The gene expression levels were normalized to the endogenous control gene 18S. N = 3; *, P < 0.05; **, P < 0.01 (chemoresistant cells *vs*. their chemosensitive cells).

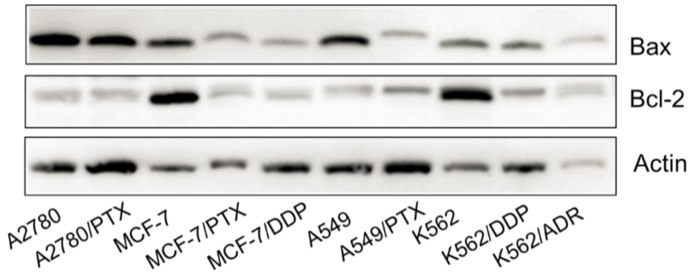

Figure S6. Expression of apoptosis-related proteins in chemoresistant cells and their chemosensitive counterparts determined by Western blot.

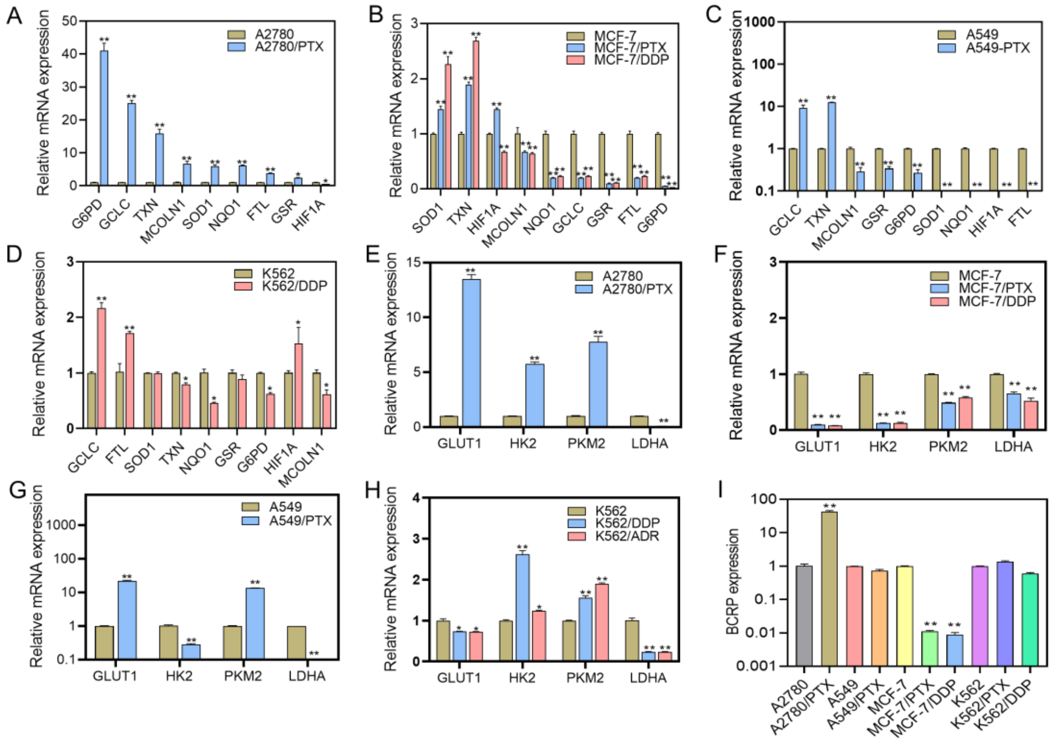

Figure S7. Relative mRNA expression of chemoresistant cells and their chemosensitive counterparts determined by qRT-PCR. (**A-D**) Expression of ROS-related genes in chemoresistant cells and their sensitive counterparts as determined by qRT-PCR. (**E-H**) Expression of glycolysis-related genes in chemoresistant cells and their sensitive counterparts as determined by qRT-PCR. (**I**) Expression of BCRP in chemoresistant cells determined by qRT-PCR. The gene expression levels were normalized to the endogenous control gene Actin. n=3; *, P < 0.05; **, P < 0.01 (chemoresistant cells *vs.* their chemosensitive cells).
